# The relationship between the expression levels of miR-135a and *HOXA10* gene in the eutopic and ectopic endometrium

**Published:** 2018-08

**Authors:** Seyed Hamidreza Mirabutalebi, Noorodin Karami, Fatemeh Montazeri, Farzaneh Fesahat, Mohammad Hasan Sheikhha, Elnaz Hajimaqsoodi, Mojgan Karimi Zarchi, Seyed Mehdi Kalantar

**Affiliations:** 1 *Genetics Department, Faculty of Medicine, Shahid Sadoughi University of Medical Sciences, Yazd, Iran.*; 2 *Abortion Research Center, Yazd Reproductive Sciences Institute, Shahid Sadoughi University of Medical Sciences, Yazd, Iran.*; 3 *Reproductive Immunology Research Center, Shahid Sadoughi University of Medical Science, Yazd, Iran.*; 4 *Research and Clinical Center for Infertility, Yazd Reproductive Sciences Institute, Shahid Sadoughi University of Medical Sciences, Yazd, Iran.*; 5 *Department of Obstetrics and Gynecology, Shahid Sadoughi Hospital, Shahid Sadoughi University of Medical Sciences, Yazd, Iran.*

**Keywords:** Endometriosis, Gene expression, Micro-ribonucleic acid, HOXA10, miR-135a

## Abstract

**Background:**

The study of microRNA expression can be effective in the diagnosing and treating different diseases. miR-135a is one of the most important micro-ribonucleic acids involved in endometriosis. Among the genes that become the target of the miR-135a and are subjected to changes in the endometrium of patients with endometriosis is *HOXA10* gene which is expressed in the endometrium in response to steroid hormones.

**Objective:**

The aim of this study was to evaluate the expression of miR-135a and its relationship with the level of *HOXA10* gene expression in both endometrial ectopic and eutopic tissues in patients with endometriosis compared to the control samples.

**Materials and Methods:**

In this prospective case-control study, both case-eutopic and case-ectopic tissue samples were obtained from 17 women with endometriosis and the eutopic endometrial tissue was sampled from 17 women with normal endometrium as the control group. The gene's expression of miR-135a and *HOXA10* were investigated using quantitative reverse transcription PCR (q-RT PCR).

**Results:**

A significant decrease in the expression of *HOXA10* gene was detected in case-eutopic during the luteal phase compared to the control samples (p=0.001), while in the case-ectopic, the expression of this gene was increased (p=0.681) compared to the control samples. In addition, the expression miR-135a in the luteal phase showed a remarkable increase in the case-eutopic endometrial tissue (p=0.026) as well as a significant decrease in the case-ectopic endometrial tissue compared to the control samples (p=0.008).

**Conclusion:**

Considering the inverse relations between the over-expression of miR-135a and the reduction of *HOXA10*, it seems that miR-135a may be applied as an endometrial diagnostic and therapeutic biomarker.

## Introduction

Endometriosis is considered as an estrogen-dependent, autoimmune and genetic disease, which is characterized by the presence of endometrial cells outside the uterus and abdominal cavity. Endometrial tissue growth outside the uterus (ectopic) in this disease is commonly seen in the abdominal cavity, pelvis (mostly on the ovaries and peritoneum) (1, 2). Approximate incidence of endometriosis in women is estimated to be about 11% in women during reproductive cycle and about 30-50% in infertile women. Also, the infertile women are 6-8 times more likely to suffer from endometriosis than healthy females (3). Despite these statistics, the exact incidence of the disease is not precisely clear, because there are no precise non-invasive tools for diagnosis of endometriosis, and in some cases it is symptomless (4). 

Ectopic transcriptome studies showed that processes such as cell adhesion, inflammation, immune system regulation, cell migration, regeneration of the extracellular matrix, and angiogenesis were associated with endometriosis (5-7). The expression of *HOXA10* gene in the normal endometrium of healthy women during the reproductive cycle has been observed in the proliferative phase and has a significantly increased expression in the luteal phase (8). In women with endometriosis, the expression of the *HOXA10* gene decreases dramatically, indicating a defect in uterine acceptance that may be responsible for reduced fertility in women with endometriosis (9). Micro-RNAs, small internal molecules with a length of about 22-24 nucleotides and non-coding proteins, are epigenetic regulators of the gene expression that are evolutionally maintained and by connecting to the 3̀UTR region of the mRNA posttranscriptional and reducing the translation levels, regulate the expression of the genes (10). 

Micro-RNAs, as a responsible factor for endometriosis pathogenesis, can be considered one of the most effective technologies for the treatment of this disease (11). The micro-RNA profile of ectopic tissues is different from that of the utopic tissues obtained from women with endometriosis (12). Micro-ribonucleic acid 135 (miR-135) was considered one of the most important micro-ribonucleic acids involved in endometriosis and also in many cancers, especially in ovarian cancer (13-15). It has been evidenced that the *HOXA10* gene is targeted by miR-135a that was increased significantly in endometriosis. So, it may a possible explanation for the reduction in the expression of this gene in women with endometriosis (16). 

So far, few studies have been conducted to evaluate the expression of miR-135a and its association with the level of *HOXA10* expression in patients with endometriosis, which indicated the relationship between the significant increase in the expression of miR-135a and the reduction of *HOXA10* expression in the endometrial eutopic tissue of patients with endometriosis. But, there is no study on the association of miR-135a with its target gene in endometrial ectopic tissue. 

The aim of this study was to evaluate the expression profile of miR-135a and its relationship with the level of *HOXA10* gene expression in both endometrial ectopic and eutopic tissues in patients with endometriosis compared to controls.

## Materials and methods

A total of 34 women with history of endometriosis who referred to Shahid Sadoughi hospital between November 2016 to MAY 2017 were enrolled in this Case-Control study. For patients were receiving Tissue from endometriotic ectopic lesions (case-ectopic, n=17) was collected during the laparoscopic session and the eutopic endometrium was obtained by endometrial biopsy in 17 women (aged 20-45 yr) (Table I). Also, endometrial tissue samples (control, n=17) from women who did not have endometriosis but referred to the hospital for some reasons such as pelvic pain or fallopian tube blockage were taken as the control samples. 

The tissues were stored in RNase free microtubes containing RNAlater™ Stabilization Solution (Thermo Fisher Scientific) at -80oC to maintain their RNA stability. The inclusion criteria were women aged 20-45 yr with regular menstrual cycles (28-32 days) and the lack of receiving a hormonal drug in the last three months. Exclusion criteria were the observation of cellular changes in the endometrium, such as hyperplasia and carcinoma, the existence of benign tumors like fibroma and polyps, and not being contaminated with common DNA viruses and the human papillomavirus as well. 

**RNA Isolation and quantitative real time-PCR**

In order to examine the expression rate of the *HOXA10* gene and miR-135a in the tissues of patients and healthy subjects, the total RNA was first extracted from the tissues using Trizol Kit (Invitrogen, USA). For eliminating the external DNA contaminations, the RNA samples were treated with DNase Ι (Fermentas). The purity and concentration of RNA were determined using spectrophotometer (NanoDrop, Thermo Fisher) and OD 260/280. Complementary DNA (cDNA) of *HOXA10* was synthesized using Revert Aid First Strand cDNA Synthesis Kit, Fermentase. Then quantitative real time-PCR (Q-PCR) was performed by 2.0 μl of constructed cDNA, 10 μl of the commercial master mix (Takara, Japan), 0.5μl of forwarding and reverse primers and 7.0 μl of DNase/RNase-free water for the genes expression profile. Glyceraldehyde 3-phosphate dehydrogenase (GAPDH) was used as the reference gene as well as *HOXA10* as the target gene (Table II). cDNA of miR-135a was synthesized using Bon-Mir RT kit (Bonyakhteh, Tehran, Iran) based on the manufacturer’s instructions. MicroRNA gene expression was carried out using thermal cycler (Applied Biosystem, ABI, Step One Plus, USA) and expression levels were evaluated using 2(-∆ct). SNORD was used as the reference gene as well as miR-135a as the target gene (Bonyakhteh, Tehran, Iran) (Table II). 

**Ethical consideration**

This study was approved by the ethics committee of Yazd Reproductive Sciences Institute, Shahid Sadoughi University of Medical Science, Yazd, Iran )IR.SSU.MEDICIN.REC.1396.16) and written informed consents were obtained from all patients. 

**Statistical analysis**

To compare the mean of the relative expression of miR-135a and its target gene *HOXA10* in three groups, SPSS software (Statistical Package for the Social Sciences, version 24.0, SPSS Inc, Chicago, Illinois, USA) and ONE WAY ANOVA with post-hoc Tukey's HSD test were used. A significant difference was considered as p<0.05. The diagrams were plotted using GraphPad Prism 7.3 software.

## Results

**The HOXA10 gene expression in the endometrium during the menstrual cycle**

Findings showed the significant increase in the expression level of the *HOXA10* gene in the endometrium of women as controls in the luteal phase of the menstrual cycle (p=0.042) compared to the follicular phase. It was observed that the expression of *HOXA10* gene has been significantly increased in endometrial lesions in the follicular phase compared with controls. In addition, a significant and remarkable reduced expression was observed in the luteal phase (p=0.001) in the eutopic tissue, and in the ectopic lesions, the expression of this gene did not have a significant difference with that of the control group in the luteal phase (p=0.681). 

**Figure 1:**
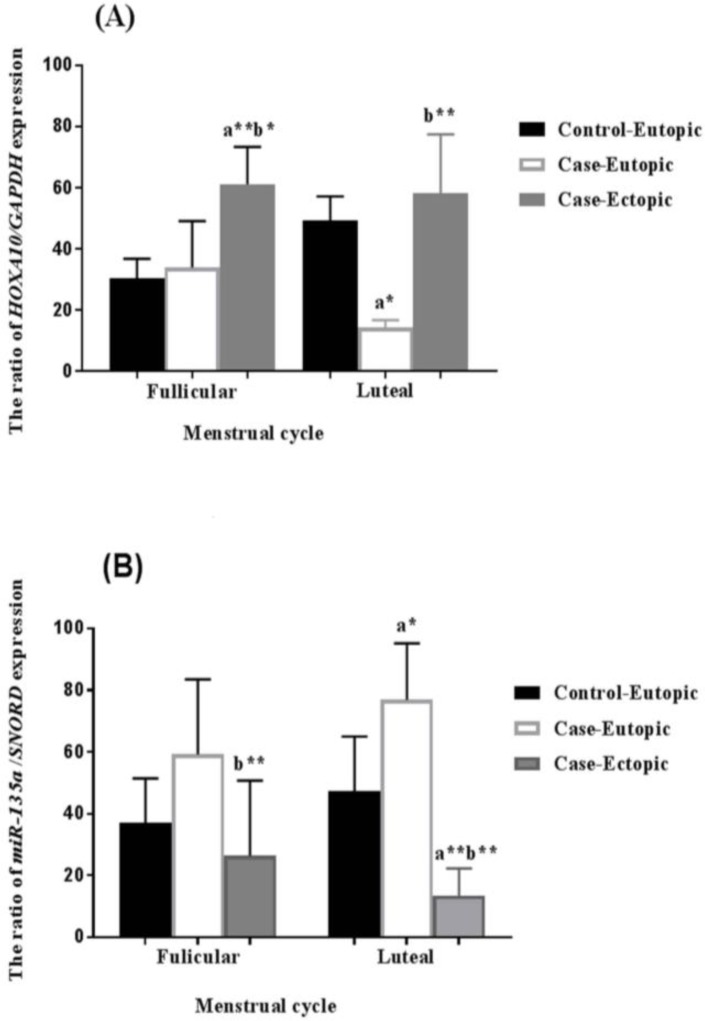
Comparing the expression of *HOXA10* and *miR-135* to *GAPDH *and* SNORD *in endometrial tissues obtained from the women with endometrium as case-ectopic and case-eutopic groups as well as women without endometrium as a control group. The ratio of gene expression of *HOXA10* to *GAPDH* (A) and the ratio of *miR-135* to SNORD (B).

According to the results, the expression of *HOXA10* gene has been altered depending on the phases of the menstrual cycle. The gene expression in the luteal phase of the menstrual cycle of the control group increased considerably compared to the follicular phase, which was statistically significant. While the gene expression in the case-eutopic group did not have a significant difference with that of the control group in the follicular phase of the menstrual cycle (p=0.991). In contrast, in the case-ectopic group, the expression of this gene was significantly increased compared to the control (p=0.001) and case-eutopic (p=0.02) groups. In the luteal phase of the menstrual cycle, the gene expression in case-eutopic reduced dramatically with respect to the control group, and in contrast, in the case-ectopic group, this gene showed a higher rate despite insignificancy compared to the control group (Figure 1A).

**The miR-135a gene expression in the endometrium during the menstrual cycle**

The findings demonstrated the expression of the miR-135a gene was unlikely to be related to the phases of the menstrual cycle. The miR-135a expression in the luteal phase and follicular phase of the menstrual cycle were not significantly different in the control group. Also, with the respect of the comparing the expression of this gene in the case-eutopic and case-ectopic endometrium of the patients with the controls, the gene expression in the case-ectopic showed a no significant difference in the follicular phase (p=0.007), while a significant increase in gene expression was observed in the case-eutopic tissue in the luteal phase (p=0.026). 

It was found that miR-135a expression in both case-eutopic and ectopic groups was not significantly different from the control group in the follicular phase. In contrast, in the luteal phase, ovarian ectopic specimens of this gene showed a significant reduction in expression compared to the case-eutopic (p=0.001) and control (p=0.008) groups (Figure 1B).

**Table I:** The demographic data of patients based on categorization in three groups

**Groups**	**Patients** **	**Menstrual cycle** **	**Age (yr)** *
Case-Ectopic	17	Follicular: 8	32 ± 2
		Luteal: 9	
Case-Eutopic	17	Follicular: 8	32 ± 2
		Luteal: 9	
Control-Eutopic	17	Follicular: 8	36 ± 2
		Luteal: 9	

* Data are presented as mean±SD.

** Data are presented as total numbers of each variable.

**Table II:** Oligonucleotide primers

**Gene**	**Primer sequence**
miR-135a	Forward: 5̀̀- GTCTCAGGGTATGGCTTTTTA- 3̀
	Reverse*
*SNORD*	Forward: 5̀̀ ATCACTGTAAAACCGTTCCA3̀
	Reverse*
*HOXA10*	Forward:5̀̀-AGGATTCCCTGGGCAATTC-3̀
	Reverse: 5̀̀-GACGCTGCGGCTAATCTCTA-3̀
*GAPDH*	Forward: 5̀̀- CTCATTTCCTGGTATGACAACGA-3̀
	Reverse: 5̀̀-TCTTCCTCTTGTGCTCTTGCTG-3̀

* Universal Reverse Primers were obtained from Bonyakhteh Company (Bonyakhteh, Tehran, Iran)

*: p<0.05, **: p<0.001, a: compared with the control group, b: compared with the eutopic group.

## Discussion

In the current study, we aimed to scrutinize the expression levels of *miR135a* in eutopic and ectopic tissues in the patients with endometriosis compared to control samples throughout the menstrual cycle and its role in the regulation of *HOXA10*. The results showed a remarkable increase in the *miR-135a* expression in the luteal phase in the case-eutopic endometrial tissue as well as a significant decrease in the case-ectopic endometrial tissue. In addition, a significant decrease in the expression of *HOXA10* gene was detected in case-eutopic samples during the luteal phase compared to the control samples, nevertheless, in the case-ectopic, the expression of this gene was increased compared to the control samples. 

Studying the relationship between the expression levels of *miR-135a* and *HOXA10* genes expression in the eutopic endometrial tissue samples in patients with endometriosis, stated that there was a significant relationship between the higher expression of *miR-135a* and decreased expression of *HOXA10* in the eutopic endometrium of the patients with endometriosis that was in agreement with our findings (16). Previous studies have proven that the expression of *HOXA10* gene in the eutopic endometrium is altered in women with endometriosis. Additionally, *HOXA10* gene was regulated directly by *miR135a *and *miR-135b *(13, 16). 

In other study conducted to examine the role of *miR-135a* tumor suppressor in epithelial tissue of the patients with ovarian cancer, it was found that *HOXA10 *gene was targeted by *miR-135a*. Further, they concluded that the expression of *miR-135a* significantly decreased in epithelial tissue of ovarian cancer, with a higher expression of *HOXA10* in contrast (13). In the same study on breast cancer findings showed that *miR-135a* represented an increased expression in the metastasis of the breast tumor (17). On the other hand, *HOXA10* gene was considered as a metastatic suppressor in breast cancer. They showed that *miR-135a* targeted *HOXA10 *gene and suppressed its expression both at the mRNA and protein levels, which our results also explained such this relationships between *miR-135a and HOXA10 *genes. Szczepańska showed the levels of *HOXA10* gene expression in the eutopic tissue of infertile women with endometriosis have significantly decreased compared with control subjects in line with ours (18).

This study showed significant differential patterns in the *miR-135a* expression profile during menstrual cycles of women either with normal endometrium or with endometriosis. Analysis of the miRNA profile in the endometriosis has improved our knowledge about the process of endometrial disease. Comparison of the ectopic and eutopic endometrium revealed the posttranscriptional patterns that have led to the formation of new perspectives in the unique cellular processes for the ectopic endometrium (12).* miRNA* regulated many different processes during the menstrual cycle, but their hormonal regulation in the endometrial functions and differentiation has still remained unknown (19). Few studies have evaluated the *miRNA* expression profile during the menstrual cycle. For instance, wang and associates reported the differential expression of *miR-142-5p* and *miRa-146-5p* in eutopic endometrium tissue during implantation window of patients with endometriosis that may cause affecting endometrial receptivity (20). 

The main challenge for the development of advanced diagnostic and therapeutic tools for endometriosis is our slight knowledge of the pathology of this disease. The genetic factors and the higher rates of estrogenic hormones increased the risk of developing endometriosis (3). The novelty point of our study is that no previous study has investigated the expression profile of the *miR-135a* in ectopic endometriotic tissue and whether the endometriotic lesions present a different *miRNA* pattern during menstrual cycles in women with endometriosis. 

Several pieces of evidence showed that epigenetic mechanisms such as microRNAs play an important role in the pathogenesis of this disease (21). The study by Andersson showed the hypermethylation rates of *HOXA1*0 gene promoter in the eutopic endometrium of the patients than the control samples. In contrast, in the ectopic endometrium of patients affected by endometriosis, few methylation rates of* HOXA10* gene promoter was detected than their eutopic endometrium of those patients, stated of the ectopic tissues contribute to a better etiopathologic understanding of endometriosis (22). Although, the role of epigenetic changes in the etiology of endometriosis has not yet been determined, the present study together with mentioned studies (18, 23) supported the hypothesis that *HOXA10* homeodomain transcription factor plays an important role in human endometrium, which is related to the success in the implantation, and also explains that *HOXA10* is necessary for denovo endometrial development. 

## Conclusion

Considering the inverse relations between the over-expression of miR-135a and the reduction of *HOXA10* expression, it is concluded that miR-135a may be applied as an endometrial diagnostic and therapeutic biomarker in the early diagnosis of endometriosis, and identification of this relation would also help to understand the etiology of endometriosis.

## Note

***This article extracted from M.Sc. thesis. (Seyed Hamidreza Mirabutalebi)***
